# Metallothionein mediates leukocyte chemotaxis

**DOI:** 10.1186/1471-2172-6-21

**Published:** 2005-09-15

**Authors:** Xiuyun Yin, David A Knecht, Michael A Lynes

**Affiliations:** 1 Department of Molecular and Cell Biology, 91 North Eagleville Rd., U-3125, University of Connecticut, Storrs, CT USA 06269-3125

## Abstract

**Background:**

Metallothionein (MT) is a cysteine-rich, metal-binding protein that can be induced by a variety of agents. Modulation of MT levels has also been shown to alter specific immune functions. We have noticed that the MT genes map close to the chemokines Ccl17 and Cx3cl1. Cysteine motifs that characterize these chemokines are also found in the MT sequence suggesting that MT might also act as a chemotactic factor.

**Results:**

In the experiments reported here, we show that immune cells migrate chemotactically in the presence of a gradient of MT. This response can be specifically blocked by two different monoclonal anti-MT antibodies. Exposure of cells to MT also leads to a rapid increase in F-actin content. Incubation of Jurkat T cells with cholera toxin or pertussis toxin completely abrogates the chemotactic response to MT. Thus MT may act via G-protein coupled receptors and through the cyclic AMP signaling pathway to initiate chemotaxis.

**Conclusion:**

These results suggest that, under inflammatory conditions, metallothionein in the extracellular environment may support the beneficial movement of leukocytes to the site of inflammation. MT may therefore represent a "danger signal"; modifying the character of the immune response when cells sense cellular stress. Elevated metallothionein produced in the context of exposure to environmental toxicants, or as a result of chronic inflammatory disease, may alter the normal chemotactic responses that regulate leukocyte trafficking. Thus, MT synthesis may represent an important factor in immunomodulation that is associated with autoimmune disease and toxicant exposure.

## Background

Initiation of an immune response is accompanied by physiological changes that can produce a stressful environment for both the cells involved in the immune response, and for bystander cells that are part of adjacent but uninvolved tissues. These stresses can be further increased by the presence of infectious microorganisms. The changes to the environment include increases in reactive oxygen and reactive nitrogen species, products of cellular metabolism, and agents that initiate apoptotic or necrotic cell death.

Cells react to stressful environments with a broad range of different homeostatic responses. These responses can include the synthesis of a host of stress response proteins, including the heat shock proteins, acute phase cytokines, and metallothionein. Metallothionein is a novel member of this type of response with a unique biochemistry and an intriguing array of physiological roles. Metallothionein is small (about 7 kDa) and extremely thiol-rich [1]. The thiols participate in complexing with divalent metal cations [2]. When metallothionein binds to essential divalent metals (e.g. zinc and copper) it may serve as a metal reservoir for apoenzymes and zinc-finger transcription regulators [3,4]. Metallothionein that is induced by other divalent metal cations (e.g. mercury, cadmium,) protects essential cellular functions [5] and enhances the survival of both cells and whole organisms that are exposed to toxic heavy metals. The thiol-rich nature of metallothionein also enables it to regulate the redox potential of cells, and thus serves as a way of indirectly regulating redox-sensitive transcription via NF-kB [6]. There are also reports that link metallothionein to a much more direct interaction with NF-kB [7,8]. Metallothionein has also been found to be released to the extracellular environment in a number of different compartments, including cell culture media, serum, urine, bronchoalveolar spaces, liver sinusoids, and inflammatory lesions [9-12]. Extracellular metallothionein has been shown to have significant immunomodulatory effects both *in vivo *and *in vitro *[13-16] however the molecular mechanism(s) of this effect have yet to be elucidated.

Leukocyte movement is an essential component of the normal response to inflammatory signals. A variety of chemotactic agents can be produced by local immune cells, damaged bystander cells, and by invading microorganisms. In aggregate, these soluble signals determine the infiltration and departure of cells that participate in the inflammation, and serve as essential regulatory components of the immune response. Stress responses alter these patterns of leukocyte trafficking in various ways. For example, psychological stress in humans has been shown to increase both the magnitude of the cellular influx at an inflammatory site and the chemotactic index of peripheral blood mononuclear cells [17]. Restraint stress in hamsters has similarly increased leukocyte trafficking and delayed type hypersensitivity responses [18]. Xenobiotics may alter leukocyte trafficking in similar ways to diminish immune competence.

We have found that metallothionein has significant chemotactic activity for both cell lines and primary leukocytes. For most of the work described in this report we have used a new assay of chemotactic cell movement. The ECIS/taxis assay is sensitive enough to detect the response of a single cell, and allows automated, real-time quantification of cell movement (21). These results suggest that cell movement in stressful environments may be influenced by the presence of metallothionein, and that this protein could be an important therapeutic target for the manipulation of inflammation *in vivo*.

## Results

**Figure 1:**
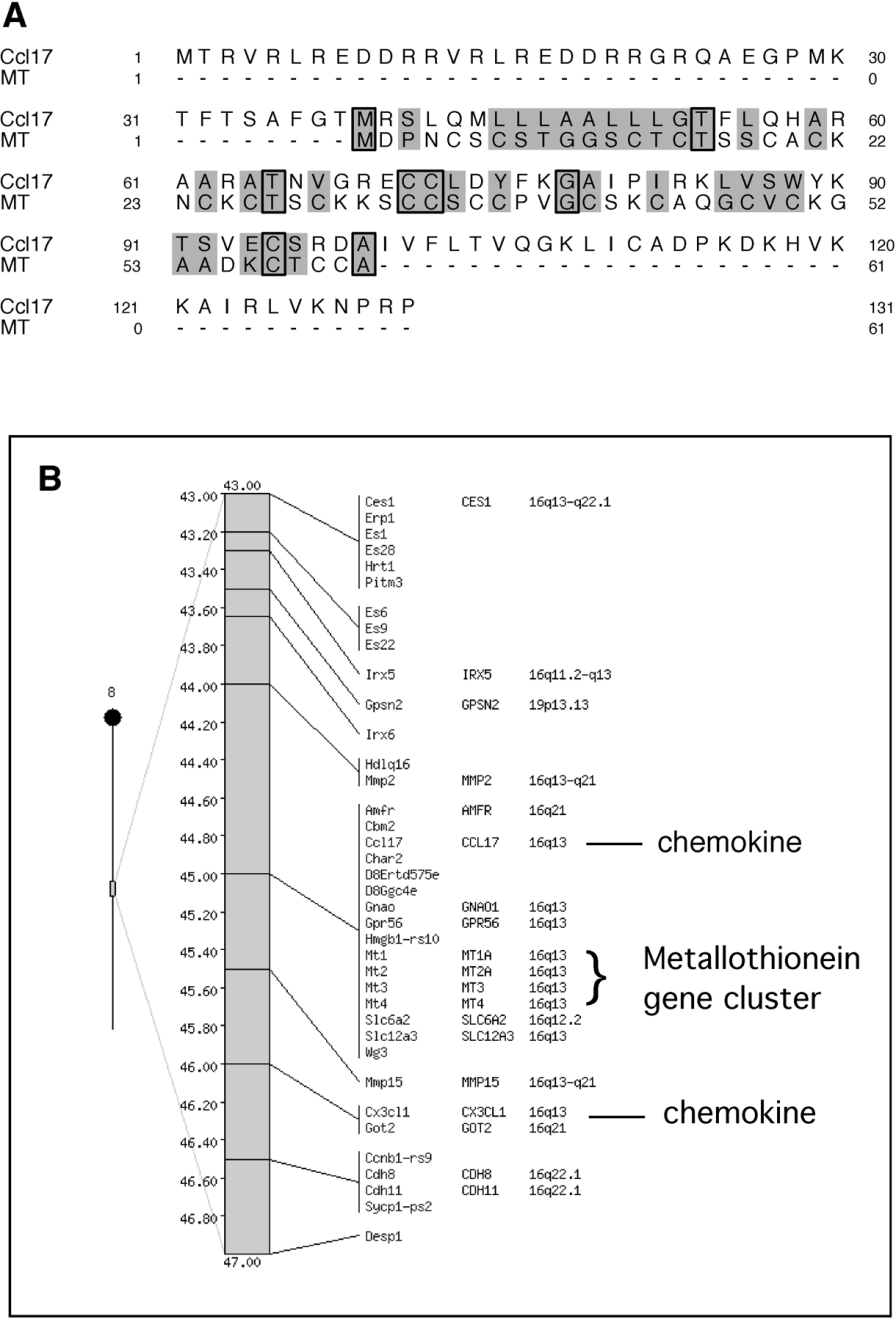
**Structural features of metallothionein gene and protein**. A: Clustal alignment of Ccl17 and metallothionein (MT) protein sequences. Amino acid similarity is set at 85% (gray). Identical amino acids are boxed. B: Mouse chromosome 8 showing expanded region from map position 43 to 47 shown on left. Homologous human genes and their chromosomal map positions are indicated on the right. Synteny map generated by the Mouse Genome Database **[63]**.

Cysteines present in the primary amino acid sequence of metallothionein are arranged in cys, cys-cys, cys-X-cys, and cys-X_3_-cys motifs. These motifs are also found in chemokine molecules, and serve to differentiate chemokine families from one another. A comparison of the sequences of metallothionein and the chemokine Ccl17 is shown in Figure 1A. In addition to having similar cysteine motifs, Ccl17 is also located near the MT genes in both mice and humans (Figure 1B). Combined with our previously published observations of the impact extracellular metallothionein has on developing T-dependent humoral immunity [15] information suggested that metallothionein might display chemotactic activity.

**Figure 2:**
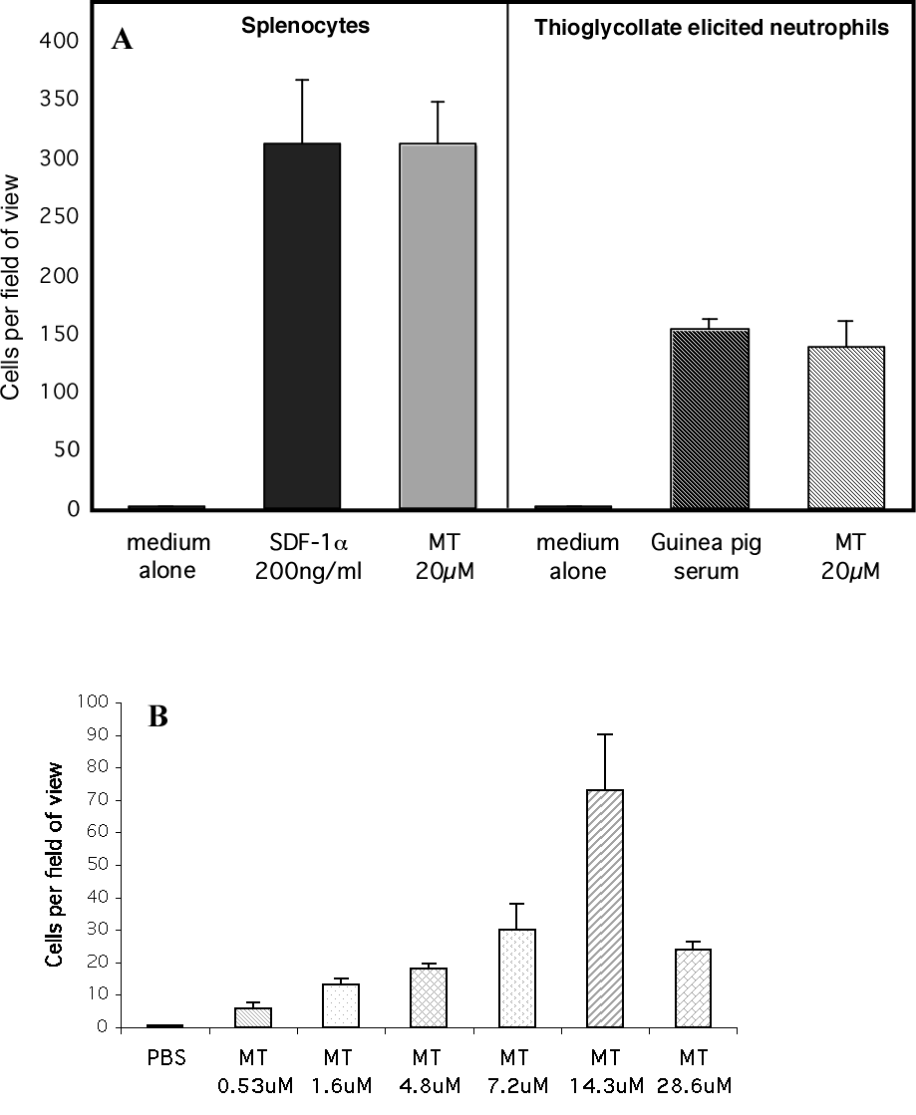
**Chemotactic responses of cells to metallothionein in the modified Boyden chamber assay**. **A. **Mouse splenocytes or Thioglycollate-elicited cells respond to guinea pig serum (as a source of activated complement) and to metallothionein. Cells that had migrated to the lower side of the filters were counted in at least six fields of view per well. Data are means of six fields in each of six replicates. The data shown is representative of three experiments and is expressed as the average ± standard deviation. **B. **Dose response curve of chemotaxis to metallothionein. Different concentrations of metallothionein were loaded into the lower wells of the Boyden chambers and Jurkat T cells were added to the upper wells.

Naïve splenocytes and thioglycollate-elicited leukocytes respond chemotactically to metallothionein in the Boyden chamber assay at a level similar to that observed when the cells are exposed to guinea pig serum used as a source of activated complement (Figure 2A). To avoid the heterogeneity of primary cell populations, the chemotactic dose response of cells to metallothionein was characterized using Jurkat T cells. Previous studies have shown that these T cells express CXCR4 (the stromal derived factor-1α (SDF-1α) receptor), and exposure to an SDF-1α gradient has previously been shown to induce a chemotactic response [19]. Using the Boyden chamber assay, we found that these cells also respond to metallothionein (Figure 2B) in a dose-dependent manner. The dose response curve shows a peak response at 14.3 μM, and the shape of the curve is consistent with that of other chemokines [20].

**Figure 3:**
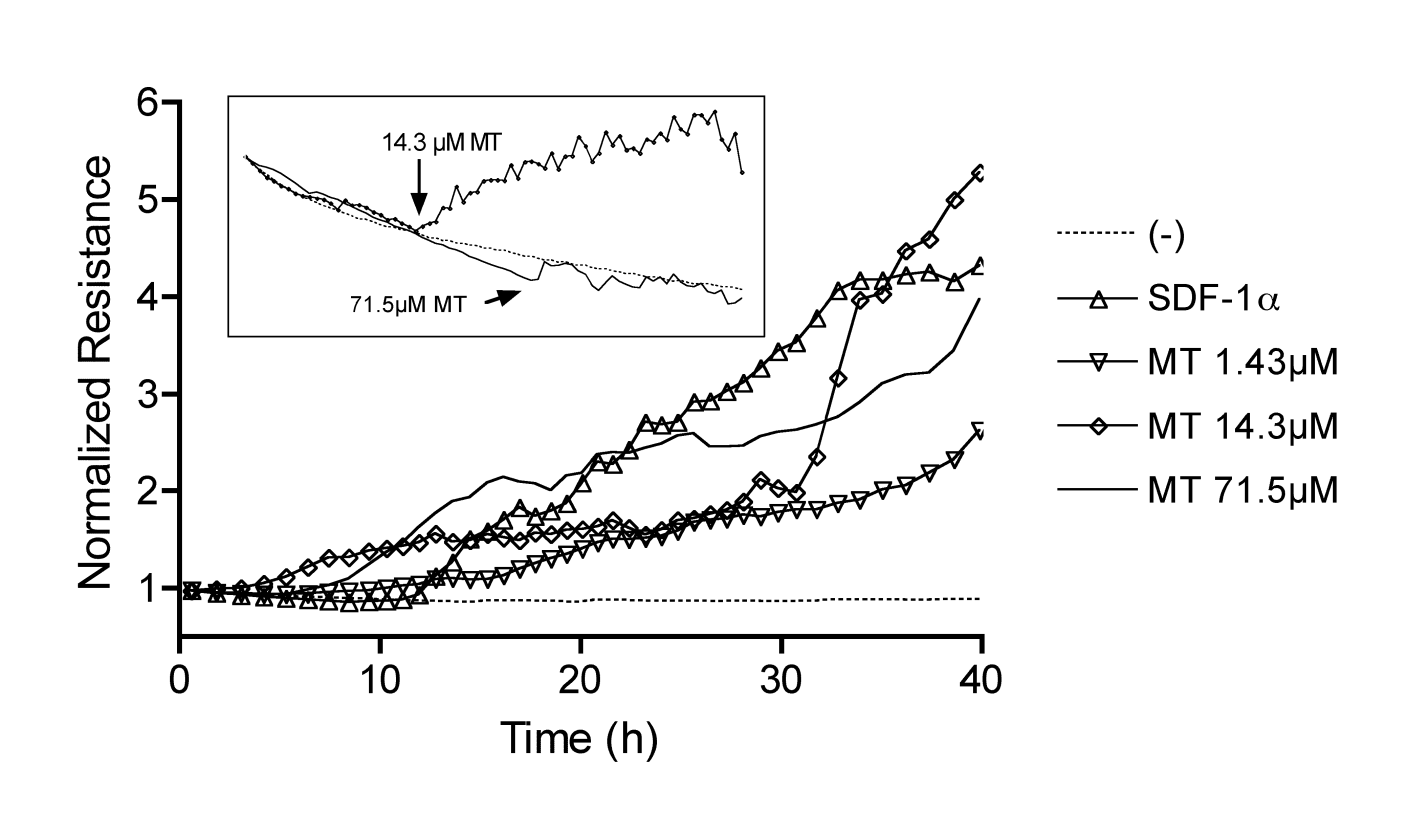
**Dose response of Jurkat T cells to metallothionein measured by ECIS/taxis**. Jurkat T cells were added to the cell wells and the indicated chemoattractant was loaded into the opposing well. SDF-1α is used as a positive control. The resistance measured at the electrode between the two wells is shown. Inset: A 3 hour window of data on an expanded scale is shown to highlight the arrival of cells on the electrode. Arrows indicated the time of arrival of the first cells leading to the appearance of small fluctuations in resistance.

**Figure 4:**
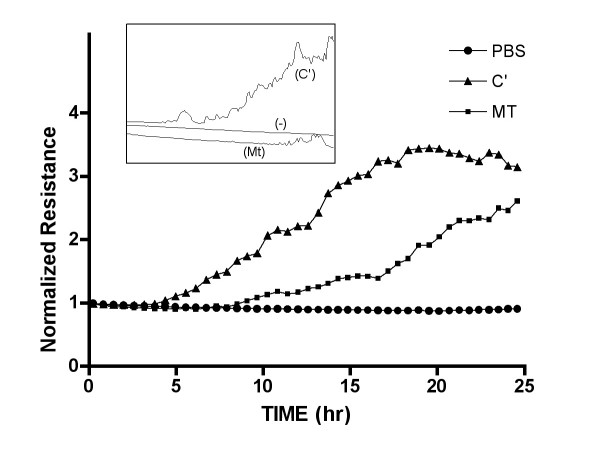
**ECIS/taxis assessment of the chemotactic response by WBC 264-9C cells**. Cell wells were loaded and guinea pig serum (1:2 dilution), PBS, or 20 μM metallothionein was added to the chemoattractant wells. Serum complement is activated by exposure to agarose to generate a control chemotactic gradient. Inset: A 3 hours window of data on an expanded scale showing the arrival of cells on the electrode.

In addition to the Boyden chamber assay, chemotaxis was assessed using a recently developed technology called ECIS/taxis. This assay employs a miniature under-agarose chemotaxis chamber in which cell arrival on a surface microelectrode is measured by changes to electrical current flow through the electrode [21]. Unlike the Boyden chamber assay, the under-agarose configuration allows the establishment of stable and shallow chemotactic gradients that more accurately reflect the subtle gradients found *in vivo *than the Boyden chamber assay. In ECIS/taxis measurements, the total normalized resistance to current flow is proportional to the number of cells that occupy the surface of the target electrode. If no chemoattractant is added, few cells move out of the well and those that do never reach the target electrode and no change in resistance is recorded (Figure 3). In the presence of a gradient of metallothionein, the initial arrival of a small number of cells at the target electrode is indicated by the appearance of small, rapid resistance fluctuations. Over time as more cells accumulate on the electrode, a gradual increase in resistance is observed. The concentration of metallothionein added to the chemoattractant well that elicited the highest resistance increase (Figure 3) and the fastest movement of responding Jurkat T cells (data not shown) was 14.3 μM. This dose optimum is similar to that measured with the Boyden chamber assay. The average speed of the fastest Jurkat T cells, 1.56 ± 0.12 μm/min, was calculated from the time of arrival of the first cells at the target electrode. This speed is comparable to the speed of the Jurkat T cell response to a gradient of SDF-1α (1.43 ± 0.1 μm/min, Figure 3). We also tested metallothionein's effect on WBC 264-9C cells. These cells have been shown to exhibit chemotactic movement toward a source of activated complement [22]. In the ECIS/taxis assay, WBC 264-9C cells are chemotactic toward both activated complement and metallothionein (Figure 4). The metallothionein response was dose-dependent, and the optimal dose was similar to that found with Jurkat T cells (data not shown). The average speed of WBC 264-9C cells responding to a gradient of guinea pig serum used as a source of activated complement was 1.32 ± 0.2 μm/min, compared to 0.75 ± 0.03 μm/min for cells responding to metallothionein. However, the population response was nevertheless robust since total resistance (indicating the absolute number of responding cells) ultimately reached a level that approximates that produced by cells responding to activated complement.

**Figure 5:**
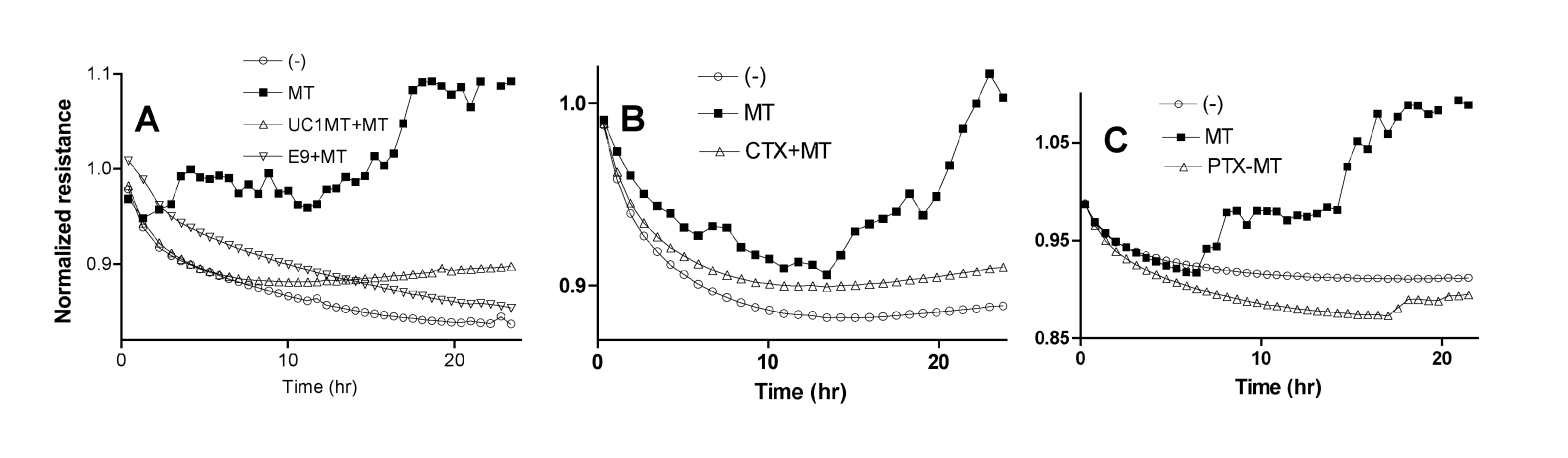
**Inhibitors of MT chemotaxis**. **A: **Monoclonal anti-metallothionein antibody blocks the chemotactic response of Jurkat T cells to 20 μM metallothionein gradient. Affinity-purified metallothionein (20 μM) was incubated with antibody (clone UC1MT or E9) and then added to the chemoattractant well. Controls were performed with metallothionein alone (MT), medium alone (-), or metallothionein preincubated with isotype-matched control antibody (not shown). **B: **Cholera toxin (CTX) inhibits chemotactic response to metallothionein. Jurkat T cells were preincubated with or without 0.133 μg/10^6 ^cells/ml CTX at 37°C for 1 h and then added to the cell well. Chemotactic responses of toxin-treated cells to MT (CTX-MT) were compared to untreated cells (MT) and untreated cells without an MT gradient (-). **C: **Pertussis toxin (PTX) blocks the chemotactic response to metallothionein. Jurkat T cells were preincubated with or without 200 ng/10^6 ^cells/ml PTX at 37°C for 16 h. Chemotactic responses of toxin-treated cells to MT (PTX-MT) was compared to untreated cells (MT) and untreated cells without an MT gradient (-). Each figure is a representative more than four independent experiments performed in triplicate.

In light of the potential for contaminants (e.g. small liver peptides that co-purify with the MT) in the commercially available metallothionein preparations, we affinity-purified metallothionein from commercial preparations using an anti-metallothionein monoclonal antibody (UC1MT) coupled to CNBr-activated Sepharose. The purified metallothionein also stimulates Jurkat T cell chemotaxis (Figure 5A). In addition, the chemotactic response to the original metallothionein (data not shown) or the affinity-purified metallothionein (Figure 5A) could be blocked by pre-incubation of the purified metallothionein with either UC1MT or E9 monoclonal anti-metallothionein antibodies. Neither of the two anti-metallothionein antibodies nor the isotype-matched IgG_1 _(MOPC 21) stimulated cell movement to the target electrode on their own (data not shown).

G protein activation has been shown to play a role in the chemotactic response and this pathway can be inhibited by cholera toxin (CTX) [23] or pertussis toxin (PTX) [24,25]. Jurkat T cells (10^6 ^cells/ml) were pre-incubated with 0.133 μg/ml CTX or 200 ng/ml pertussis toxin and then placed in a metallothionein gradient. The chemotactic effect of metallothionein on Jurkat T cells could be blocked by CTX (figure 5B) and by PTX (figure 5C). PTX was also capable of blocking the chemotaxis of Jurkat cells to SDF-1α.

**Figure 6:**
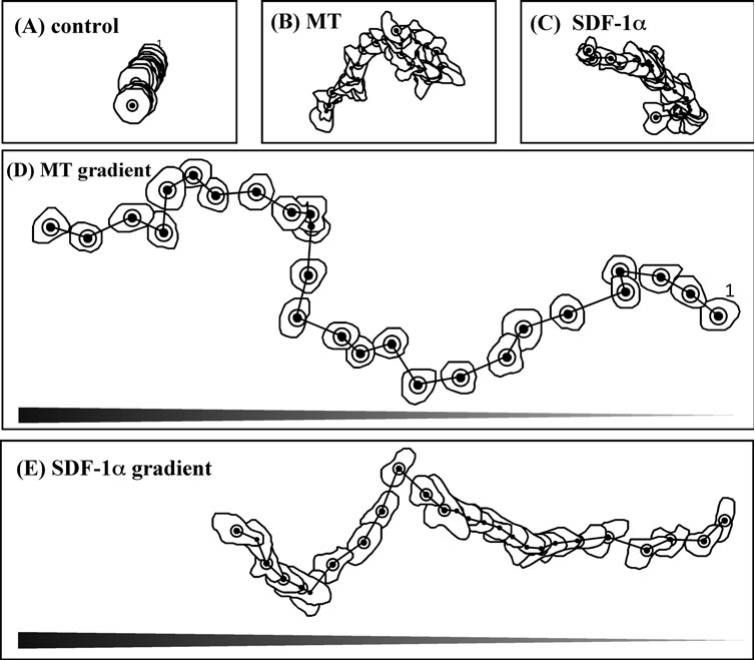
**Cell movement in the presence and absence of metallothionein and SDF-1α**. Images at 3 minute intervals (over a 75 minute time period) are presented for untreated cells (A), cells treated with a uniform concentration of MT(0.5 μM) (B), or SDF-1α (100 ng/ml) (C), or cells moving under agar in a chemotactic gradient of MT (20 μM) (D) or SDF-1α (200 ng/ml) (E). Each panel has the same number of images and represents the same total time interval. The line at the bottom of panels D and E represents the direction of the chemoattractant gradient from high (wide) to low (narrow). Only cells that have exited the cell well and are clearly visible under the agarose were analyzed.

A more direct assessment of chemotaxis was done using time-lapse video microscopy of cells moving in the presence of a metallothionein or SDF-1α gradient. When Jurkat T cells were exposed to a gradient similar to that present in the ECIS/taxis assay, they could be observed to move out of the cell well and continue up the gradient toward the chemoattractant well. Tracks of the outlines of these cells show persistent directional movement (Figure 6D, E). In the absence of a gradient few cells exit the cell well (data not shown), and those that do show little directional movement (Figure 6A–C). The speed, persistence and chemotactic indices of individual cell movements are consistent with the speeds calculated using the ECIS/taxis measurements of population movement (Table 1). In order to assess the role of chemokinesis in this process, cells were overlaid with a pre-formed agarose sheet containing a uniform concentration of metallothionein, SDF-1α or medium alone. The MT-exposed cells moved more rapidly than control cells, but the movement lacked directional persistence and was much slower than movement in a spatial gradient (Table 1 and Figure 6A–C). Similar results were obtained using a checkerboard analysis of cell movement in the Boyden chamber format (Table 2). Metallothionein added to the same side of the filter as the cells, or to both sides of the filter in equal concentration resulted in fewer cells reaching the lower surface of the filter than in wells where the metallothionein was added to the opposite side of the filter from the cells. This data supports the conclusion that metallothionein induces both chemotaxis and chemokinesis in Jurkat T cells.

**Figure 7:**
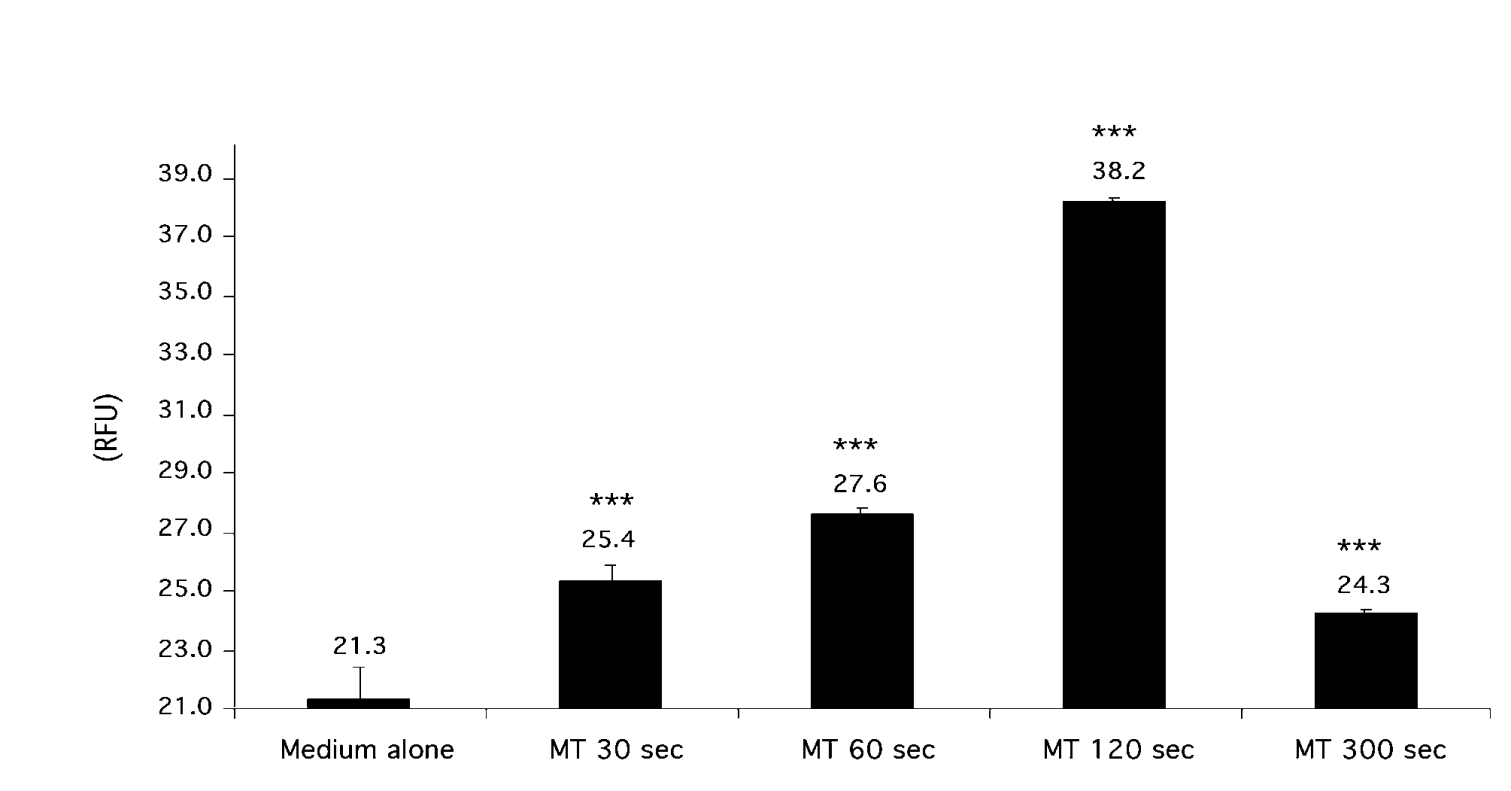
**Actin reorganization in metallothionein-stimulated cells**. Jurkat T cells were treated with metallothionein at 2 μM and then harvested and fixed at various times after stimulation. Cells were stained with rhodamine phalloidin and F-actin fluorescence quantified using a plate fluorimeter. (RFU = relative fluorescent units). Values reported represent the average of 3 replicates ± standard deviation. *** represents significant difference from the medium alone control (p < 0.001). This data is representative of 3 independent experiments.

Another hallmark of cellular responses to chemokines is a change in the amount and distribution of polymerized actin. Signal transduction through G protein coupled receptors causes reorganization of the actin cytoskeleton, leading to the formation of new F-actin rich lamellipods that extend in the direction of movement. This reorganization can be assessed by *in vitro *measurements of polymerized actin from cell extracts of stimulated cells with phalloidin [26]. Metallothionein stimulated a 19% increase in total F-actin within 30 seconds and a 79% increase by 2 minutes (Figure 7). The extent and timing of this response is consistent with receptor activation in other cell types [27,28].

## Discussion

Cells of the immune system operate in a complex microenvironment where they are presented with a host of different and often conflicting signals [29,30]. The ways in which cells integrate and respond to these signals can ultimately govern the way in which the immune system will respond to antigen exposure. In some cases, the outcome is an activated immune response that is designed to eliminate the source of antigen. In other instances, the cells become anergic or undergo apoptosis and thus fail to initiate or participate in an immune response to that antigen. An early aspect of many immune responses is the directional movement of cells toward a site of infection or other injury. This directional movement is a response to chemotactic factors produced by some infectious organisms, to chemokines produced by cells already at the site of inflammation, or to other agents. Cells that express receptors for these signals can detect the gradient(s) of diffusing chemoattractants, and move toward the source of the agent. This chemotactic response is an essential aspect of lymphocyte trafficking.

In this report, we show that metallothionein can direct the chemotaxis of primary and transformed leukocytes. While metallothionein has been historically thought of as an intracellular protein, there are numerous reports that describe its presence in serum, urine [31], broncho-alveolar spaces [10], liver sinusoids [32], and other extracellular locations. While the mechanism(s) by which metallothionein is released from cells has yet to be determined, heat shock protein 70 [33], Interleukin 1β [34] and fibroblast growth factor [35] are among a set of proteins that lack signal sequences and nevertheless are released from cells by a non-traditional secretory mechanism. These results indicate that stress response proteins may gain access to the extracellular environment via mechanisms other than cell lysis, and suggest that a thorough understanding of the immunomodulatory roles played by metallothionein must include the extracellular compartment.

Metallothionein is synthesized in response to acute phase cytokines (e.g. IL-1, IL-6, and TNF-a) that are secreted at sites of inflammation [36-38] in a variety of contexts in which immune activities are changing. Metallothionein is also synthesized in cells exposed to glucocorticoids, a signal that is often associated with stressful environments [39]. Furthermore, metallothionein can be induced by reactive oxygen species, by endotoxin [40], and in cells exposed to divalent metal cations [1]. With all of these different initiators, it is not surprising that elevated metallothionein levels are detected in the context of neoplastic disease [41,42], autoimmune disease [43], chronic inflammation [44], and infection [45]. Previous work from our laboratory and others has shown that metallothionein can have significant immunomodulatory activities. For example, metallothionein can diminish T dependent humoral responses and it can alter the proliferative capacity of lymphocytes [16], diminish cytotoxic T cell function [46], and it can alter the effector function of macrophages [47]. Inadequate expression of metallothionein in the context of inflammatory disease can dramatically shorten life span [43], and exogenous metallothionein can diminish the severity of a collagen-induced arthritis [48].

There are a multitude of studies which show that different forms of stress originating from external sources can alter normal immune function [49]. Psychological, physical and chemical agents which induce stress each affect the immune system. In some instances, these stressors suppress effective immune functioning, which renders the individual susceptible to infectious pathogens. In other instances, the immune modifications result in undesirable increases in immune recognition of self antigens, ultimately resulting in autoimmune disease. These stressors are known to induce metallothionein synthesis and may alter immune functions in part via their effect on metallothionein.

We have demonstrated metallothionein-induced chemotactic cell movement in the traditional Boyden chamber assay, by computerized analysis of time-lapse images of cell movement, and using the ECIS/taxis assay. We have shown that the response of Jurkat T cells to a metallothionein gradient corresponds well with chemotactic responses of leukocytes to other agents. Jurkat T cells and WBC 264-9C migrate in response to a metallothionein gradient at speeds which are similar to those found in other systems [50,51]. In addition, the pattern of the dose response to metallothionein is a bell shaped curve similar to other classical chemokines [20]. One important consideration is whether the chemotactic response to metallothionein occurs at physiologically relevant concentrations. Chemoattractants can act over an extremely wide concentration range (e.g. 4 logs) [52,53] because the cells sense the local spatial differential in chemoattractant concentration. Our work shows that 1 to 10 μM metallothionein can stimulate chemotaxis of cells in both Boyden and under-agarose assays. Higher concentrations of metallothionein used in the ECIS/taxis assay refer to the concentrations added to the micro-volume chemoattractant wells, which are then diluted in the process of diffusion away from the source. The metallothionein amounts used in these experiments represent biologically reasonable concentrations, given that metallothionein has been measured at concentrations of 1 μM in serum (which would be substantially diluted from the source tissue concentration) in normal patients, and in individuals undergoing some form of stress (inflammation, cancer, toxicant exposure, etc.) [54].

The chemotactic response to metallothionein can be blocked by monoclonal antibodies to metallothionein while isotype-matched antibody has no effect. This blockade of the response is an important control, since commercial metallothionein preparations contain contaminating peptides from the liver tissue source (D. Lawrence, personal communication). Since both cholera toxin and pertussis toxin block the metallothionein-initiated chemotaxis, it is likely that G protein mediated signaling is involved in the response. Another common aspect of chemotactic signaling is an activation of the actin polymerization machinery in response to a sharp increase in chemoattractant concentration [26,55]. Metallothionein causes an increase both in total F-actin content and in peripheral F-actin. It will be of great interest to determine the receptor for metallothionein and the signal transduction pathway that leads to actin polymerization.

It is intriguing to speculate that once outside the cell, metallothionein serves as one of the many signals designed to draw immunocompetent cells to sites of cellular stress. Our observation(s) that metallothionein and anti-metallothionein injections modify immune activity *in vivo *suggest that there is an appropriate range of extracellular metallothionein in which leukocytes ordinarily function [14,15]. A pair of recent reports suggest that cytosolic constituents of apoptotic cells are released to the extracellular compartment and support the progression of the inflammatory process [56,57]. These reports further suggested that release of the cytosolic components of these dying cells might represent one of the signals central to the "Danger Hypothesis" proposed by Matzinger *et al. *[58,59]. This hypothesis holds that an active immune response cannot be mounted without a signal indicating that cellular damage has occurred. While other reports have suggested that heat shock proteins can fill this role [60], metallothionein is another potential candidate for the danger signal.

## Methods

### Cells

Jurkat T cells, (TIB-152, American Type Culture Collection (ATCC), Bethesda, MD) were maintained in complete RPMI 1640 media with L-glutamine containing 10% heat-inactivated FBS (Mediatech, Herndon, VA), 1% Sodium Bicarbonate, 100 units/ml penicillin, 0.1 mg/ml streptomycin, 1% sucrose, and 1 mM sodium pyruvate as recommended by ATCC. WBC 264-9C cell lines (HB-8902, ATCC) were kept in Minimum Essential Medium (Eagle) with Earle's balanced salt solution (BSS) containing 10% heat-inactivated fetal bovine serum. All cells were cultured in a humidified incubator with 5% CO_2 _in air at 37°C. The WBC 264-9C is a macrophage-like cell line that is chemotactic to N-formylmethionyl-leucyl-phenylalanine [61]. Media was replenished every three days.

### Reagents

SDF-1α (Synthetic Human SDF-1α) was purchased from BD Biosciences (Bedford, MA). BSA (DNase, RNase, and Protease-free) and Hema-3 stain set kit were purchased from Fisher Scientific Inc. (Pittsburgh, PA). A mixture of Cd, Zn-metallothionein I and II purified from rabbit liver, mouse IgG_1, _kappa (MOPC21) purified immunoglobulin, and pertussis toxin, were purchased from Sigma Chemical Co. (St Louis, MO). SeaKem^® ^GTG^® ^Agarose was obtained from BioWhittaker Molecular Applications (Rockland, ME). Metallothionein monoclonal antibodies UC1MT (IgG_1_, kappa) [14,15], available from StressGen, Inc., Victoria, BC and E-9 (IgG_1_, kappa), purchased from Zymed Laboratories Inc. (San Francisco, CA) were used in some experiments. Cholera toxin was purchased from List Biological Laboratories, Inc. (through Cedarlane, Ltd., Hornby, ONT Canada). Guinea pig serum was purchased from Colorado Serum Company (Denver, CO).

### Affinity purification of metallothionein

UC1MT was first purified on ProteinG-Sepharose (Sigma) according to manufacturer's instructions. The purified antibody was then coupled to CNBr-activated Sepharose (Sigma) according to manufacturer's instructions. Metallothionein I and II, prepared in PBS, was mixed with the immobilized UC1MT and allowed to bind. After unbound proteins were washed away from the affinity matrix, the specifically captured protein was eluted with 0.1 M glycine HCl, pH 2.8, adjusted pH to 7.4 and dialyzed against PBS.

### Boyden chamber assay

The micro-Boyden assay was done using a 48 well chamber apparatus (NeuroProbe, Cabin John, MD). Polyvinylpyrrolidone (PVP)-free polycarbonate membrane filters with 5 μm pores were obtained from the same source. The lower chambers of the apparatus were loaded with 30 μl of diluted chemoattractant in media, PBS vehicle in media, or media alone and then covered with the membrane and the upper chambers. Fifty microliters of cell suspension (2 × 10^6 ^cells/ml) was then added to the upper chambers. After incubating for 2 hours in a humidified incubator at 37°C in 5% CO_2_, the filters were collected, cells that remained on the upper surface of the filter were removed and the filters were processed according to manufacturer's instructions. The numbers of migrated cells were counted under 400× magnification. For each of six replicate wells, the numbers of cells in at least six fields were determined and the mean and standard deviation was calculated.

### ECIS/taxis assay

This assay was done as previously described with minor modifications [21]. Linear electrode ECIS chambers (Applied Biophysics, Inc. Troy, NY) were used in the assays described here. Target electrodes were 0.02 × 2 mm, and were used in an orientation in which the long axis of the target electrode was oriented perpendicularly to the direction of cell migration. All the chambers containing electrodes were pre-treated with 10 mM cysteine for 15 min at room temperature to stabilize the electrical performance of the gold electrodes, washed three times with sterile distilled water, and dried in a standard biosafety laminar-flow hood. Then 250 μl of molten 0.5% agarose gel (dissolved in RPMI 1640 with 10% FBS) was added to each chamber and allowed to cool. Two wells were cut with a sharpened 14 gauge cannula equally distant on either side of the electrode and separated a combined intrawell distance of approximately 1.9 to 2 mm. Then 7 μl of cell suspension (15 × 10^6 ^cells/ml for Jurkat T cells and 10 × 10^6 ^cells/ml for WBC 264-9C cells) was placed into the cell well and an equal volume of chemoattractant or vehicle control was dispensed into the opposite well. A 1 volt AC current of 4000 Hz is passed through the electrode, and the resistance of the circuit was calculated. Cell movement was assessed by measurements of changes in the resistance caused by arrival of cells at the target electrode. Data is reported as the change in resistance at the target electrode normalized to the initial resistance of the system. In addition to the general increase in resistance caused by cells covering the electrode, rapid fluctuations in resistance are indicative of changes in the shape and surface adherence of cells, and of continuing cell viability and movement.

### Trough chemotaxis assay

For some chemotaxis experiments,3.5 ml of 0.5% agarose (dissolved in medium with 10% FBS and 20 mM HEPES) was loaded into a 35 mm Petri dish. After the agarose solidified, 2 wells separated by about 2 mm were cut in the agarose. One well was loaded with 7 μl of chemoattractant or media and the opposing well was loaded with 7 μl of cell suspension. The Petri dish was then sealed with Parafilm to retain moisture and incubated on the microscope stage at 37°C. Temperature was maintained by enclosing the microscope in a Styrofoam box in which a constant temperature airstream was provided by an Air-Therm feedback regulated heater (WPI, Inc., Sarasota FL). Chemokinesis in the under-agarose environment was investigated by seeding cells to the surface of the Petri dish in liquid media. After the cells had settled, the overlying media was removed and the cells were overlaid with a pre-gelled layer of agarose containing a uniform concentration of the different stimuli or medium alone. Images of the cells were taken at regular intervals using a CCD-72 analog video camera (Dage, Michigan City, IN) and Scion frame grabber controlled by Scion Image (Scion, Inc., Frederick, Maryland) software. The images were compiled into movies using public domain Image J software [62]. The trajectories of cells were analyzed from these movies using Dynamic Image Analysis System (DIAS) software (Solltech, Inc., Oakdale, IA). Trajectories of a number of cells from each condition (see n in table 1) were tracked and analyzed from the movies. Each cell was tracked for the same total time interval and the data is presented as the mean of all cells analyzed.

Measurements of actin polymerization: Cells were spun at 200 × g for 5 minutes and resuspended in RPMI 1640 containing 10% FBC at a density of 2 × 10^6 ^cells/ml. Cells were stimulated with either SDF-1α (data not shown) or 2 uM metallothionein at 37°C and then fixed with 3.7% formaldehyde for 15 minutes in Buffer F on a rotator at room temperature (5 mM KCl, 138 mM NaCl, 4 mM NaHCO_3_, 0.4 mM KH_2_PO4, 1.1 mM Na_2_HPO_4_, 2 mM MgCl_2_, 2 mM EGTA, 5 mM PIPES, pH 7.2). The fixed cells were centrifuged and resuspended in 1 ml of 0.5% Triton X-100 in Buffer F for 20 minutes and stained in 1 uM TRITC-Phalloidin in Buffer F on a rotator for 1 hour at room temperature. They were then pelleted, washed with 5 ml Buffer F twice, and re-suspended in 850 μl of Buffer F. The TRITC-Phalloidin fluorescence in the cell suspension was measured using a Spectramax M2 fluorimeter (Molecular Devices, Sunnyvale, CA) at 544 nm excitation and 580 nm emission. In each well, the raw fluorescence of 9 points were measured and used to calculate the average well fluorescence.

## Authors' contributions

XY designed and carried out all of the experiments drafted the manuscript. DAK and MAL conceived of the study, and participated in its design and coordination and helped to author the manuscript. All authors read and approved the final manuscript.

## Figures and Tables

**Table 1:** DIAS analysis of chemokinetic and chemotactic movement. Jurkat T cells were cultured under agarose in the presence of uniform concentrations or in a gradient of the stimuli. Cells were imaged over time and their motile behavior quantified. Persistence is an indicator of the rate of directional change. Chemotactic index is a measure of the proportion of movement in a designated direction (1 = toward a source, 0 = random movement, -1 = away from the source).

**Stimulus (n = number of analyzed cells)**	**Average translational speed (μm/min)**	**Persistence (μm/minute-degree)**	**Chemotactic index**
Media control (7)	0.66 ± 0.29	0.18 ± 0.11	0.19
Uniform concentration of metallothionein (10)	2.110 ± 0.73	0.5 ± 0.22	0.07
Uniform concentration of SDF-1α (10)	1.69 ± 0.82	0.53 ± 0.34	0.09
Metallothionein gradient (7)	6.6 ± 3.2	2.042 ± 1.09	0.73
SDF-1α gradient (7)	3.31 ± 1.48	1.17 ± 0.49	0.72

**Table 2:** Checkerboard analysis of chemokinetic cell movement induced by metallothionein. Metallothionein has both chemokinetic and chemotactic activities. Significant chemotactic movement is measured when metallothionein is presented from below the filter. Chemokinetic movement is indicated when metallothionein is present above the filter, or in both chambers. The data is representative of three independent experiments.

	Cell number migrated to lower side of membrane			
	
	Metallothionein (μM) above filter			
Metallothionein (μM) below filter	0	2.5	5.0	10

0	14.0 ± 4.3	90.8 ± 17.5	43.0 ± 3.2	20.6 ± 9.2
2.5	55.3 ± 17.0	61.3 ± 23.9	38.7 ± 12.5	25.5 ± 13.2
5.0	192.8 ± 45.4	93.6 ± 20.1	67.0 ± 12.5	37.0 ± 12.1
10	80.2 ± 23.7	96.0 ± 21.8	68.0 ± 21.2	30.8 ± 10.3
